# Nonrecurrent inferior laryngeal nerves and anatomical findings during thyroid surgery: report of three cases

**DOI:** 10.1186/s40792-016-0170-5

**Published:** 2016-05-17

**Authors:** Kumiko Kato, Yasuo Toriumi, Makiko Kamio, Hiroko Nogi, Hisashi Shioya, Hiroshi Takeyama

**Affiliations:** Department of Breast and Endocrine Surgery, The Jikei University School of Medicine, 3-25-8, Nishi-shinbashi, Minato-ku, Tokyo, 105-8461 Japan

**Keywords:** Nonrecurrent inferior laryngeal nerve, Thyroid surgery, Aberrant right subclavian artery, Vagus nerve

## Abstract

A nonrecurrent inferior laryngeal nerve (NRILN) is found more frequently on the right side than on the left, and it is closely associated with an aberrant right subclavian artery. The presence of the aberrant right subclavian artery on preoperative computed tomography (CT) scan suggests NRILN; however, different types of branching locations and pathways exist. Here, we report three NRILN cases with different pathways where the vagus nerve arises more medial than usual and a review of the literature. Case 1: A 30-year-old Japanese female presented with papillary thyroid carcinoma. Preoperative CT scan revealed an aberrant right subclavian artery, and an operation was performed under suspicion of NRILN. During the operation, the vagus nerve was found to arise more medially than usual and two NRILNs originated from it at the level of the cricoid cartilage and at a more caudal position; the two NRILNs were preserved. Case 2: A 33-year-old Japanese female with a thyroid nodule of increased size underwent surgery. Preoperative CT scan revealed an aberrant right subclavian artery, which suggested NRILN. During the operation, the vagus nerve was identified to run more medially than usual and NRILN was found to originate at the level of the cricoid cartilage; NRILN was preserved. Case 3: A 78-year-old Japanese female underwent an operation with a diagnosis of papillary thyroid carcinoma. Preoperative CT scan showed an aberrant right subclavian artery. During the operation, NRILN was found to originate from the vagus nerve at the level of the lower pole of the thyroid gland, and the vagus nerve ran medial to the common carotid artery at the caudal level.

## Background

In cervical operations, including those for thyroid disease, injury of the recurrent inferior laryngeal nerve is one of the serious complications. Large tumors, locally advanced cancer, and anomalies of the recurrent laryngeal nerve may cause an intraoperative nerve injury. The following have been reported as anomalies of the recurrent laryngeal nerve: nonrecurrent nerve [1–5], bifurcation into anterior and posterior branches before entering the larynx [6], and medial malposition on the right side due to the right aortic arch [7, 8]. Among them, nonrecurrent inferior laryngeal nerve (NRILN) is commonly caused by an embryologic anomaly of the aortic arch, such as an aberrant right subclavian artery. As a result of dysplasia, recurrent laryngeal nerve does not recur in the mediastinum and originates from the vagal trunk at a specific location of the neck [9]. The presence of an aberrant right subclavian artery on preoperative computed tomography (CT) scan suggests NRILN; however, there are various types of branching locations and pathways. Here, we report three cases of NRILN with different pathways where the vagus nerve runs more medially than usual, and we review the pertinent literature.

## Case presentation

### Case 1

**Fig. 1:**
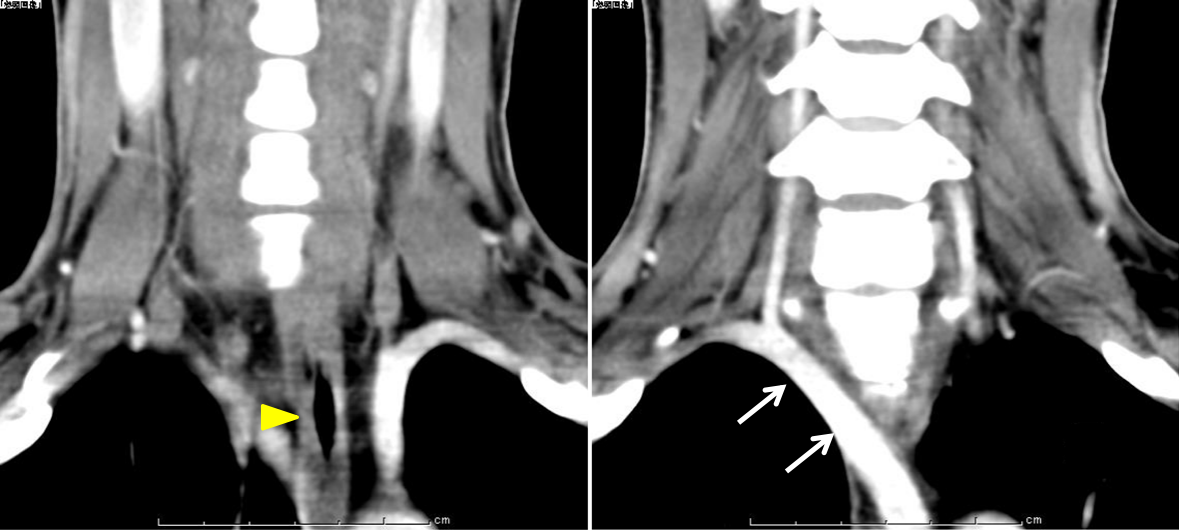
Preoperative enhanced CT scan of neck and chest of case 1. A right subclavian artery (*white arrow*) was found to originate from the aorta left of the midline and ran to the right axilla region crossing behind the esophagus (*yellow arrowhead*)

**Fig. 2:**
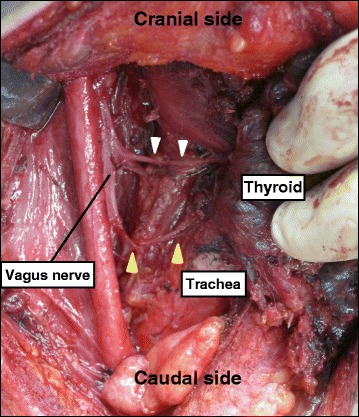
Intraoperative findings of case 1. A nerve originated from the vagus nerve at the level of the cricoid cartilage and entered the larynx (*white arrowhead*), and another nerve to the larynx originated at the caudal level (*yellow arrowhead*). The vagus nerve was located more medial than usual

A 30-year-old Japanese female presented to a clinic with a right neck mass; it was diagnosed as a thyroid nodule. This patient was referred to our hospital, and ultrasound examination revealed a homogeneous and isoechoic mass of 25 mm in diameter in the right upper lobe, a hypoechoic mass of 9 mm with high-echoic spots behind it, and several swollen lymph nodes. Contrast-enhanced CT showed an aberrant right subclavian artery (Fig. 1). Fine-needle aspiration cytology revealed papillary carcinoma. Based on the above findings, the patient was diagnosed with papillary carcinoma of the thyroid (T2N1aM0, stage I) and she underwent total thyroidectomy and neck dissection. After reaching the thyroid by collar incision, pre- and para-tracheal lymph node dissection was performed on the left side; the left recurrent laryngeal nerve was identified as running normally. On the right side, however, as loose connective tissue superficial to the right common carotid artery was incised, it was found that the vagus nerve was located medial to the common carotid artery. Furthermore, two ramifications from the nerve were identified: one originated at the level of the cricoid cartilage and entered the larynx, and another originated at a more caudal level and entered the larynx. Both nerves were thought to be branches of NRILN. A branch from the vagus nerve to the esophagus was also identified, and all these nerves were preserved (Fig. 2). The postoperative course was uneventful, and the patient was discharged from the hospital on the ninth postoperative day. She did not complain of any symptoms associated with recurrent laryngeal nerve palsy. The histopathological examination revealed follicular type of papillary carcinoma.

### Case 2

**Fig. 3:**
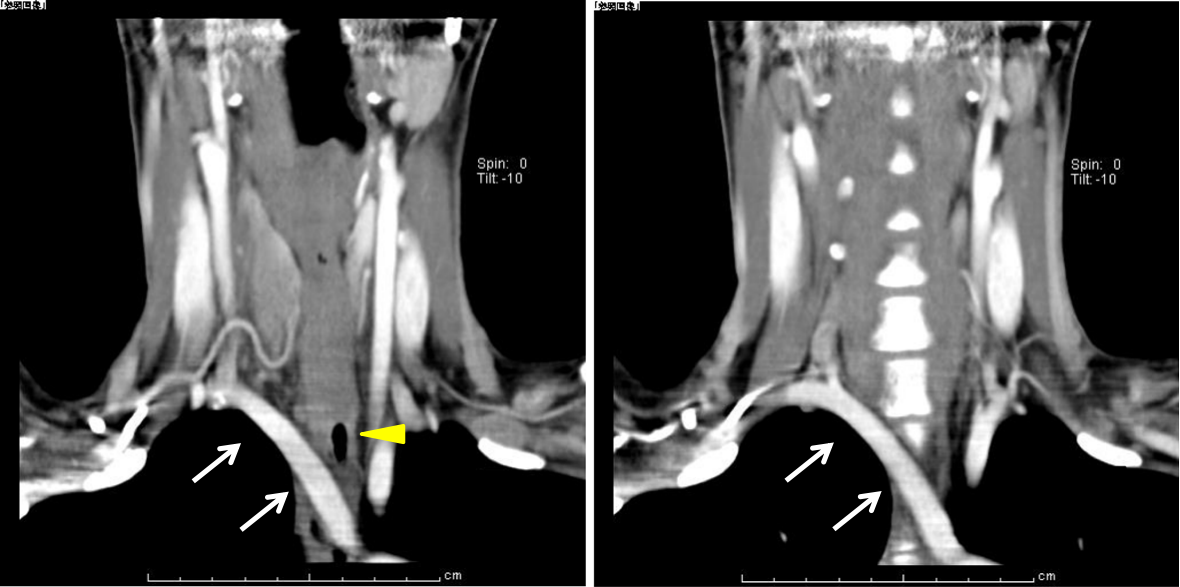
Preoperative enhanced CT scan of neck and chest of case 2. An aberrant right subclavian artery (*white arrow*) was found crossing behind the esophagus (*yellow arrowhead*)

**Fig. 4:**
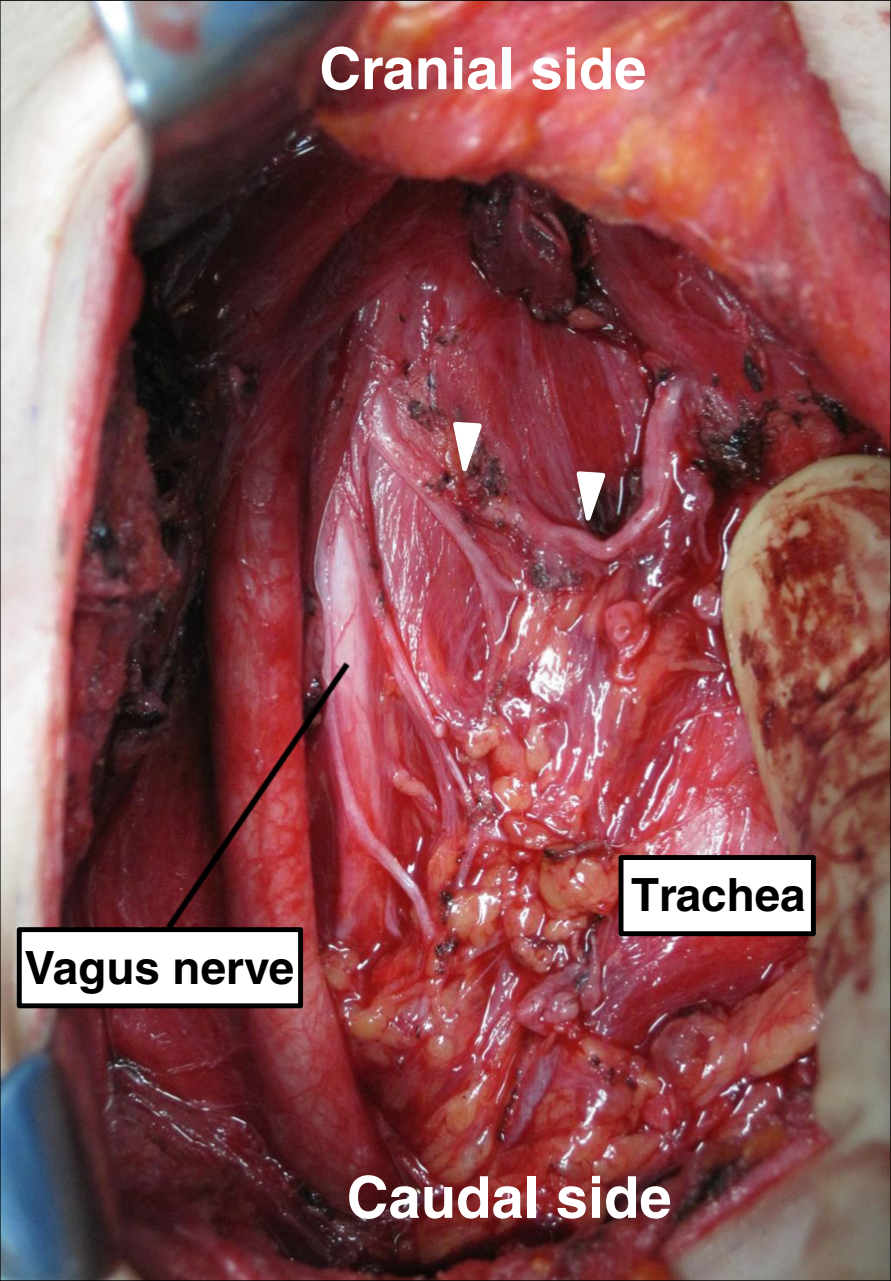
Intraoperative findings of case 2. A nerve originated from the vagus nerve at the level of the cricoid cartilage and entered the larynx (*white arrowhead*). The vagus nerve was located more medial than usual

A 33-year-old Japanese female presented with a slow-growing nodule in the right lobe of the thyroid gland. Ultrasound examination of the thyroid revealed a solid lesion of 40 mm in diameter extending from the isthmus to both lobes. Contrast-enhanced CT revealed a 40-mm nodule with heterogeneous enhancement mainly in the thyroid isthmus and an aberrant right subclavian artery (Fig. 3). The result of fine-needle aspiration cytology was benign. Subtotal thyroidectomy was performed due to the thyroid nodule size. After reaching the thyroid by collar incision, the left side was dissected and the left recurrent laryngeal nerve was identified to be in the normal position. On the right side however, as loose connective tissue superficial to the right common carotid artery was incised, it was subsequently found that the vagus nerve was medial to the common carotid artery. Furthermore, a ramification from the vagus nerve was identified to originate at the level of the cricoid cartilage and to enter the larynx; the nerve was considered to be a laryngeal branch of NRILN. At the caudal location, several branches to the esophagus were found (Fig. 4). All these nerves were carefully preserved throughout the procedure. Postoperative complication such as recurrent laryngeal nerve palsy was not observed, and the patient was discharged on the seventh postoperative day. The histopathological examination revealed follicular adenoma of the thyroid.

### Case 3

**Fig. 5:**
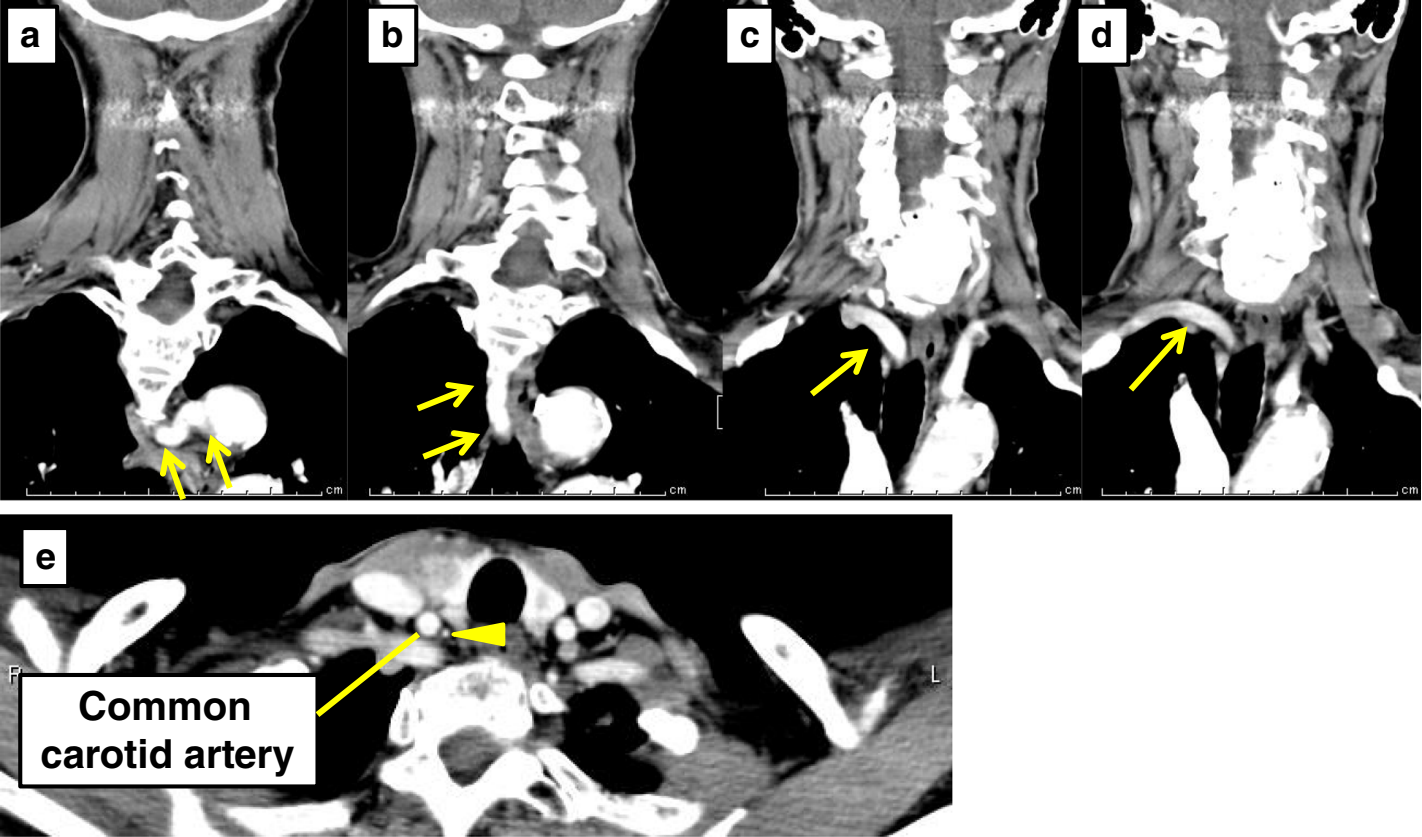
Preoperative enhanced CT scan of the neck and chest of case 3. **a**–**d** CT revealed an aberrant right subclavian artery (*yellow arrow*). **e** An artery ascending medial to the common carotid artery was thought to be a right vertebral artery (*yellow arrowhead*)

**Fig. 6:**
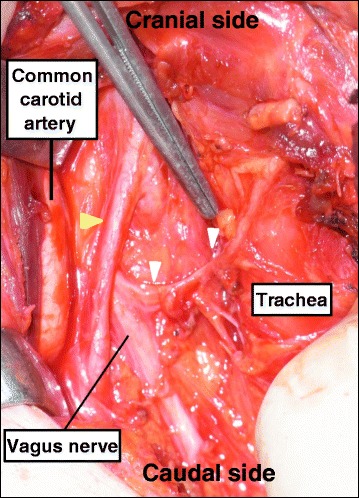
Intraoperative findings of case 3. A nerve originated from the vagus nerve at the level of the lower pole of the thyroid and entered the larynx (*white arrowhead*). At a caudal site, the vagus nerve was located medial to the common carotid artery. Also, an artery ascending medial to the common carotid artery was identified (*yellow arrowhead*)

A 78-year-old Japanese female presented with a right neck mass at our hospital. Ultrasound examination revealed a solid lesion of 35 mm in diameter in the right lobe of the thyroid and several swollen lymph nodes in the right neck. Contrast-enhanced CT scan showed a 32-mm nodule with heterogeneous enhancement in the right lobe and an aberrant right subclavian artery (Fig. 5a–d). Fine-needle aspiration cytology revealed papillary carcinoma. From the above findings, we diagnosed the nodule as papillary carcinoma of the thyroid (T2N1aM0, stage III) and she underwent total thyroidectomy and neck dissection. After reaching the thyroid, the intervention made was from the left side of the neck and the left recurrent laryngeal nerve was identified to be in the normal position. On the right side, while incising the loose connective tissue superficial to the right common carotid artery, an artery ascending medial to the common carotid artery was identified (Fig. 6). By reconfirmation of CT scan images during surgery, a possible anomaly of the right vertebral artery was noted; it originated from the subclavian artery at the same position as the origin of the right common carotid artery and ascended medial to the common carotid artery (Fig. 5e). NRILN, a ramification from the vagus nerve branching out at the level of the lower pole of the thyroid and entering the larynx, was identified and preserved carefully. After separating the NRILN, the vagus nerve was found to descend medial to the common carotid artery at the caudal level. Postoperative complication such as recurrent laryngeal nerve palsy was not observed, and the patient was discharged from the hospital on the seventh postoperative day. The histopathological examination revealed papillary carcinoma of the thyroid.

### Discussion

In 1823, Stedman first proposed a postmortem case of NRILN. In 1932, a case with NRILN was found during cervical surgery and it was reported by Pemberton and Beaver [1]. NRILN drew the attention of surgeons and was known to be a pitfall during surgery. The reported incidence of NRILN has ranged from 0.3 to 0.52 % of cervical surgery [10–12], while the presence of NRILN on the left side is extremely low, 0.04 % [11]. Generally, the point of discussion is about the presence of NRILN on the right side. Right-sided NRILN is closely associated with an aberrant origin of the right subclavian artery, which is one of the common aortic malformations known as a developmental anomaly accompanied by the right NRILN [2, 9]. In a case with an aberrant right subclavian artery, the right subclavian artery arises more distal than the left subclavian artery and reaches the right upper arm towards the retro-esophagus. This explains the fact that the right recurrent laryngeal nerve originates from the vagus nerve at the level of the neck.

An understanding of the position and the pathway of NRILN originating from the vagus nerve is necessary to avoid injury to the nerve during cervical surgery. NRILN types have been classified based on the relationship with the thyroid gland [11] and thyroid arteries both superior and inferior [10, 12]. Henry et al. reported that NRILN was identified in 33 cases (0.52 %) out of 6307 cervical operations; specifically, 31 were found as the right NRILN and 2 were as the left one [11]. The level of the origin of NRILN was at the upper pole of the thyroid in 29 cases, and at the middle-to-lower pole of the thyroid in 4 cases; thus, most NRILN origination sites shown were at the upper thyroid pole. Avisse et al. divided the NRILN into two types; in type 1, NRILN originates from the level of the upper thyroid pole to the larynx, while in type 2, NRILN originates and runs parallel to the inferior thyroid artery and then ascends to the larynx. Avisse et al. also reported seven cases of type 1 and 10 cases of type 2 among 17 cases of the right NRILN [10]. Toniato et al. encountered 31 cases (0.51 %) of the right NRILN out of 6000 cervical operations, including five cases in type 1 and 26 cases in type 2 [12]. Moreover, they proposed a subdivision of type 2 into two subtypes; one crosses posterior to the interior thyroid artery and the other passes between the branches of the artery. The latter two reports included frequent cases of NRILN originating from the vagus nerve which was closely related to the inferior thyroid artery. These findings suggest that NRILN can be identified at the level between the upper thyroid lobe and the branching site of the thyroid artery.

In the present three cases, preoperative CT revealed the aberrant subclavian artery and we were able to predict the presence of NRILNs. These NRILNs originated from the vagus nerve at an upper level in two cases and at a lower location in one case. Moreover, in the three cases, the vagus nerve descending medial to the common carotid artery and NRILN originating from the vagus were rather easily identified after incising the loose connective tissue superficial to the common carotid artery. In our clinical department, such incising of the loose connective tissue superficial to the common carotid artery is a routine procedure during thyroid surgery. The vagus nerve may have shifted medially as a result of the incision of the carotid artery in our three NRILN cases; however, such medial shifts of the vagus nerve have never been observed in other surgical cases. Thus, we reached a conclusion that cases of NRILN would be originally accompanied by the medially shifted vagus nerve. To the best of our knowledge, no case report has described the NRILN’s medial shift of the vagus nerve. Furthermore, case 3 presented an anomaly of the vertebral artery, which might have been caused by an aberrant subclavian artery. This could suggest the importance to pay attention to not only the nerves but also the vessels when an aberrant subclavian artery is present. More importantly, two branches entering the larynx were recognized as NRILN in case 1. Henry et al. argued variations of branches in 33 right NRILN cases. Among them, NRILN had two branches to the larynx in 3 cases [11]. Including our case, these cases might correspond to the bifurcation into anterior and posterior branches before entering the larynx of the normal inferior laryngeal nerve. Thus, it is necessary to always keep such a variation in mind during thyroid surgery.

## Conclusions

In conclusion, we have encountered three cases of NRILN which originated from the vagus nerve running medial to the common carotid artery and showed different pathways. It is necessary to predict the existence of NRILN by preoperative CT scan, and surgery should be performed allowing consideration for many possible branching variations of NRILN. Furthermore, when encountering a vagus nerve which runs medial to the carotid artery during an operation, it is important to be reminded of the existence of NRILN even without recognizing the presence of an aberrant right subclavian artery.

### Consent

Written informed consent was obtained from the patient for publication of this case report and any accompanying images. A copy of the written consent is available for review by the Editor-in-Chief of this journal.

## Abbreviations

CT: computed tomography
NRILN: nonrecurrent inferior laryngeal nerve

## References

[1] Pemberton J, Beaver MG (1932). Anomaly of the right recurrent laryngeal nerve. Surg Gynecol Obstet.

[2] Hermans R, Dewandel P, Debruyne F, Delaere PR (2003). Arteria lusoria identified on preoperative CT and nonrecurrent inferior laryngeal nerve during thyroidectomy: a retrospective study. Head Neck.

[3] Sanders G, Uyeda RY, Karlan MS (1983). Nonrecurrent inferior laryngeal nerves and their association with a recurrent branch. Am J Surg.

[4] Yalxin B (2006). Anatomic configurations of the recurrent laryngeal nerve and inferior thyroid artery. Surgery.

[5] Obaid T, Kulkarni N, Pezzi TA, Turkeltaub AE, Pezzi CM (2014). Coexisting right nonrecurrent and right recurrent inferior laryngeal nerves: a rare and controversial entity. Report of a case and review of the literature. Surg Today.

[6] Sugino K, Okamoto H, Kataoka T, Yano M, Okajima M, Asahara T (2003). Clinical study of early extralaryngeal division of recurrent laryngeal nerve. 6th Japanese Res Soc Clin Anatomy Meeting Abstract Book.

[7] Sato S, Tachibana S, Yokoi T, Yamashita H (2012). A case of papillary thyroid carcinoma with right aortic arch—relation with recurrent laryngeal nerve and aortic arch anomaly. J JAES JSTS.

[8] Knight L, Edwards JE (1974). Right aortic arch: types and associated cardiac anomalies. Circulation.

[9] Sadler TW (2010). Langman’s medical embryology.

[10] Henry JF, Audiffret J, Denizot A, Plan M (1988). The nonrecurrent inferior laryngeal nerve: review of 33 cases, including two on the left side. Surgery.

[11] Avisse C, Marcus C, Delattre JF, Marcus C, Cailliez-Tomasi JP, Palot JP (1998). Right nonrecurrent inferior laryngeal nerve and arteria lusoria: the diagnostic and therapeutic implications of an anatomic anomaly. Surg Radiol Anat.

[12] Toniato A, Mazzarotto R, Piotto A, Bernante P, Pagetta C, Pelizzo MR (2004). Identification of the nonrecurrent laryngeal nerve during thyroid surgery: 20-year experience. World J Surg.

